# Echocardiographic Predictors of Improvement of Left Ventricular Ejection Fraction below 35% in Patients with ST-Segment Elevation Myocardial Infarction

**DOI:** 10.3390/jcm13144016

**Published:** 2024-07-09

**Authors:** Hezzy Shmueli, Gal Tsaban, Anna Moreno, David Shamia, Itai Weissberg, David Uziel, Artyom Star, Khaled Elhaj, Yigal Abramowitz

**Affiliations:** Cardiology Department, Soroka University Medical Center, Faculty of Health Sciences, Ben Gurion University of the Negev, P.O. Box 151, Beer Sheva 84101, Israelyigalab@yahoo.com (Y.A.)

**Keywords:** ST-segment elevation myocardial infarction, echocardiography, ejection fraction, left ventricle recovery

## Abstract

**Introduction**: An ST-elevation myocardial infarction (STEMI) is a clinical syndrome defined by symptoms of myocardial ischemia, persistent electrocardiographic ST-segment elevation and subsequent release of biomarkers suggestive of myocardial necrosis. In clinical practice, echocardiography has become essential in evaluating patients after acute myocardial infarction. We aimed to assess clinical and echocardiographic parameters that may affect LV function recovery in patients after STEMI. **Methods**: This study is a retrospective observational study from a tertiary referral center in Israel. We collected patients that were admitted with STEMI and a left ventricular ejection fraction (LVEF) below 35% on preliminary echocardiography at the index hospitalization and compared the findings to a follow-up study performed within 1–6 months after that event, in order to see if there are predictors of LVEF change > 10% within 90 days following STEMI. **Results**: This study included 101 patients that were admitted between 2016 and 2021. Within a median follow-up of 9.7 weeks (IQR 5.9–17.1), 27 (25.2%) patients had improved their LVEF, and 74 (69.2%) had no change or further reduced LVEF. Compared to patients without LVEF improvement, those with improved LVEF were more likely to be female (29.6% vs. 9.5%, *p* = 0.01), less likely to suffer from hypertension (33.3% vs. 56.8%, *p* = 0.04) and had marginally higher rates of thrombolysis treatment (14.1% vs. 4.1%, *p* = 0.06). **Conclusions**: in the population of STEMI patients with residual LVEF < 35%, approximately a quarter will improve at least 10% in their follow-up LVEF, and there were no clear echocardiographic predictors for this improvement.

## 1. Introduction

ST-elevation myocardial infarction (STEMI) is a clinical syndrome defined by symptoms of myocardial ischemia in association with persistent electrocardiographic ST-segment elevation and subsequent release of biomarkers suggestive of myocardial necrosis [1]. The prognosis of STEMI patients depends on several parameters, among them the infarct’s size, collaterals presence and time to revascularization of the occluded artery [2]. STEMI remains a significant contributor to morbidity and mortality worldwide, despite a declining incidence and better survival rates [1,2,3]. Left ventricular (LV) dysfunction due to acute infarction leading to myocardial necrosis and remodeling can identify patients at higher risk of sudden cardiac arrest and death [4,5,6] and remains an important predictor of morbidity and mortality even in an era of primary percutaneous coronary intervention (PCI). Improvement of LV function may occur early after MI due to recovery of hibernating or stunned myocardium; however, the degree of long-term LV recovery is tempered by adverse LV remodeling from myocyte necrosis, inflammation and fibrosis [4].

In clinical practice, echocardiography has become an integral component in evaluating patients after acute myocardial infarction (MI). Various echocardiographic parameters have been shown to provide prognostic information in this population, such as LV volumes and LV ejection fraction, wall motion score index, mitral regurgitation and left atrial volume. The introduction of tissue Doppler imaging and speckle-tracking strain imaging has resulted in additional prognostic parameters [7]. Speckle tracking echocardiography permits the assessment of myocardial strain in three spatial directions (longitudinal, radial and circumferential) independent of the ultrasound beam angle and is expressed as a percentage, defined as the relative change in length/thickness of the LV myocardium concerning its original length/thickness [8]. In addition, global strain was found to be a marker of arrhythmias in post-MI patients even with otherwise normal parameters of LV function [9]. Global longitudinal strain (GLS) provided incremental prognostic information beyond the Framingham risk score, the SCORE risk chart and the modified ACC/AHA pooled cohort equation for the composite outcome and incidence of heart failure (HF) after STEMI [10]. Nonetheless, all these parameters mentioned above were not evaluated for their association with LV function recovery post-MI.

As congestive heart failure (CHF) in STEMI patients is known as a significant cause of morbidity and mortality, identifying patients with high risk to develop CHF may aid in selecting more appropriate post-infarction therapies and additional care and management. However, many of the previous studies concerning this topic occurred before the institution of modern HF therapies, rapid revascularization techniques and novel LV echocardiographic assessment tools, which may attenuate the inferences of their findings. Existing data provide limited prognostic value to help us understand the HF risk profile of patients following an acute MI event. Moreover, there is no known solid association between immediate post-MI echocardiographic parameters and improvement in LV function post-MI.

Therefore, the purpose of this study was to assess clinical and echocardiographic parameters that may affect LV function recovery in patients after STEMI that were treated according to the acceptable guidelines.

## 2. Materials and Methods

### 2.1. Study Design and Population

This study is a retrospective observational study. Our Cardiac Intensive Care Unit (CICU) in Soroka University Medical Center in southern Israel is a large, tertiary (and practically the only) referral center for over 1 million people, including remote areas where thrombolysis therapy is used whenever necessary. We built a database based on ICD-9 and ICD-10 admission and discharge codes to our CICU between 2016 and 2021. The medical and echocardiographic data were derived anonymously from the medical records of patients.

### 2.2. Inclusion Criteria

- Patients who were admitted to the Soroka University Medical Center CICU between the years 2016 and 2021.
- Diagnosis of STEMI.
- Had successful primary PCI (with or without initial thrombolytic therapy).
- Had an echocardiographic LV function of ≤35% during the index hospitalization, and a follow-up echocardiography during a 1–6 months period after the index STEMI hospitalization.

### 2.3. Echocardiography Data

- All echocardiography studies were performed either in our institution or in large community referral clinics associated with Clalit health services, which is the largest health provider in Israel. All studies were performed by experienced sonographers and were interpreted by senior cardiologists specialized in echocardiography. All studies were performed with VIVID E3 95 ultrasound device manufactured by GE and were held following standard protocol of the ASE guidelines.
- Echocardiographic variables that were examined were the following: LV end diastolic diameter, LV end systolic diameter, interventricular septum (IVS), left ventricle posterior wall (LVPW), fractional shortening (%), LA (left atrium)-diameters, LA-AREA, LA-VOLUME, RA-superior inferior, RA-AREA, left ventricle ejection fraction (LVEF) estimated quantitively by the interpreter or by GLS or by the Simpsons’ method, 2D global longitudinal strain, mitral flow [E wave, A wave, E/A ratio, deceleration time, E/e’], mitral regurgitation (MR), tricuspid regurgitation (TR), aortic regurgitation (AR), estimated right atrium (RA) pressure, estimated systolic pulmonary artery pressure, right ventricle (RV) function, LVOT (LV outflow tract diameter), LVOT-velocity time integral, aortic annulus diameter, ascending aorta diameter, diastolic function, pericardial fluid, RV diameter at base, RV diameter at mid and wall motion abnormalities—any, anterior, inferior/posterior, septal, lateral and apical.

#### 2.3.1. Primary Outcome

Echocardiographic predictors of left ventricular ejection fraction change > 10% within 90 days following ST-segment elevation MI.

#### 2.3.2. Secondary Outcome

Clinical parameters that are predictors of left ventricular ejection fraction change > 10% within 90 days following ST-segment elevation MI.

### 2.4. Ethics

The study protocol was approved and was held following the institutional IRB committee.

## 3. Statistical Analysis

Baseline characteristics and exposure variables were compared across LVEF improvement groups with a cut-off of at least 10% improvement. Categorical variables are presented as percentages and tested using the chi-squared test or Fisher’s exact test if expected rates > 5 were more than 30%. Continuous variables are presented as means and standard deviations or medians and interquartile ranges, as appropriate. Continuous variables were compared using Student’s independent *T*-test or Mann–Whitney U-test, as appropriate. A logistic regression model was used to predict recovery of at least 10% in ejection fraction compared to the initial echo study during the index hospitalization. Due to the study’s sample size, we first created a propensity score depicting the probability having a recovery of at least 10% in ejection fraction. All potential confounders were tested for collinearity, which was not detected. Variables included in the propensity score-adjusted logistic regression were entered based on clinical relevancy and/or statistical significance (entry criteria *p* < 0.10 in univariate analysis). The final propensity score model included the following variables: age, sex, ethnicity, diabetes mellitus, hypertension, congestive heart failure, prior myocardial infarction, smoking status, obesity and days of follow-up. Next, separate models were constructed for each exposure variable of interest, adjusting for the propensity score only and therefore maintaining model stability. Continuous predictors or nonlinear associations with each outcome variable in unadjusted models were interrogated. The results of the models are presented as odds ratios (ORs) with 95%CI. A two-sided *p*-value of less than 0.05 is considered statistically significant for all analyses. Statistical analyses were conducted using SPSS software version 28.0 (Armonk, NY, USA).

## 4. Results

**Figure 1:**
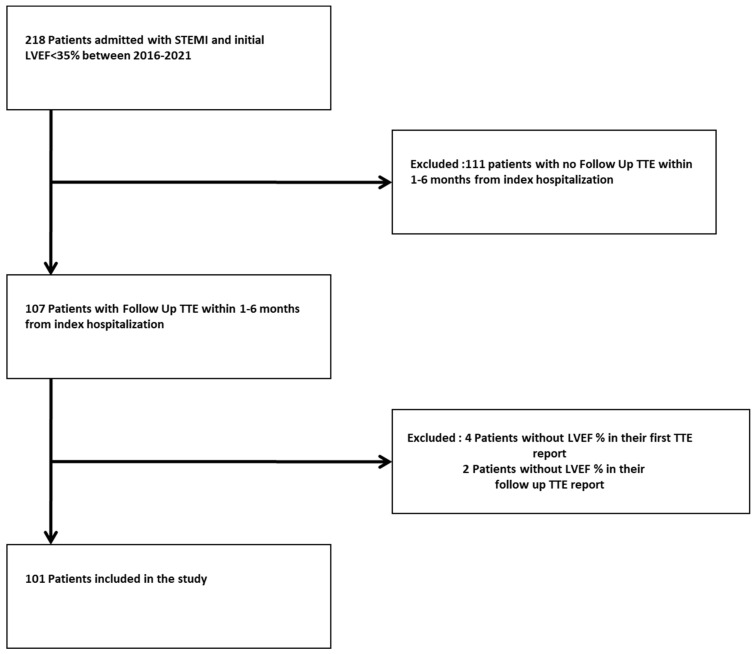
Patient selection and inclusion flow chart.

The final study cohort included 101 patients who had validated baseline and follow-up transthoracic echocardiography left ventricular ejection fraction estimations. The flow chart that shows this process is shown in Figure 1. During a median follow-up of 9.7 weeks (IQR 5.9–17.1), 27 (25.2%) patients had improved their LVEF, and 74 (69.2%) had no change or further reduced LVEF.

The baseline characteristics of the study population are detailed in Table 1, and echocardiographic findings of the patients across LVEF improvement status are detailed in Table 2. Briefly, patients had a median age of 63 (IQR 52–72) and were more likely to be of Jewish ethnicity (77.2%). Compared to patients without LVEF improvement, those with improved LVEF were more likely to be female (29.6% vs. 9.5%, *p* = 0.01), less likely to suffer from hypertension (33.3% vs. 56.8%, *p* = 0.04) and had marginally higher rates of thrombolysis treatment (14.1% vs. 4.1%, *p* = 0.06). None of the patients with improved LVEF had chronic renal failure, as opposed to 16.2% of the patients without improved LVEF (*p* = 0.026). As for the echocardiographic data, patients had a median LVEF of 30% (IQR 22.5–35) and a global two-dimensional longitudinal strain of −10 (−11, −9). Three patients (3.0%) had moderate or above mitral regurgitation, and the systolic pulmonary pressure was normal and similar across groups (34, IQR 28–40). All patients had wall motion abnormalities, and two left ventricular thrombi were diagnosed in the acute phase. There were no significant differences between groups across various echocardiographic parameters, including LVEF. Patients in the non-improved LVEF group had numerically lower rates of pericardial effusion than those who improved (11.1% vs. 23.0%, *p* = 0.1).

Laboratory data from index hospitalization and main medical treatment are detailed in Table 3. Patients with improved LVEF had lower measured total, direct; and indirect bilirubin levels and lower maximal high sensitivity troponin levels than those without improvement (*p* = 0.01). NT-proBNP levels were not measured among those who did not improve but only among those who eventually improved. Both study groups were similarly treated. Beta-blockers and renin–angiotensin–aldosterone system inhibitors or blockers were initiated in nearly 90% of the patients. Statins were also initiated among most patients (over 95%), while spironolactone was administered to 39 patients (37%). SGLT2i therapy was initiated among 16 (15%) patients and across groups.

At follow-up, none of the patients had significant mitral regurgitation, and one patient from the improved LVEF group developed severe tricuspid regurgitation. Also, five patients were diagnosed with left ventricular thrombi.

After propensity score adjustment, only higher baseline LVEF and higher total bilirubin at presentation were associated with lower risk of LVEF rebounding by more than ten percent (see Table 4).

Data regarding the primary catheterization itself were also collected and analyzed. We hypothesized that an early and complete revascularization in STEMI may contribute to later LVEF improvement in our study population. However, as shown in Table 5, no specific parameter (such as door to balloon time, culprit coronary artery, staged procedure, etc.) was found to be correlated to our research cut-off of above 10% improvement of LVEF.

## 5. Discussion

The most important finding in this study is that, in the population of STEMI patients with residual LVEF < 35%, approximately only a quarter will improve at least 10% in follow-up LVEF. In addition, the severity of LVEF decrease following the STEMI event was not associated directly with improvement in future follow-up LVEF.

Several studies have discussed such related issues. Brooks et al. found that 57% of STEMI patients with LVEF < 35% improved compared to follow-up echo 90 days after the index hospitalization [5]. They also found several parameters that were correlated to LVEF recovery > 35% such as hospital length of stay, history of MI, lateral wall motion abnormalities and elevated troponin levels, but LVEF in admission had shown a strong and independent association with recovery to LVEF > 35%. These findings also correlated with previous data that indicated that only about 8% of this population needed sudden cardiac death primary prevention ICD transplantation with similar LVEF post-STEMI [11]. In a large Korean study, there were similar results, as 51% of instances of at least moderate LV systolic dysfunction post-STEMI have shown LVEF improvement at a mean follow-up period of approximately 220 days [12]. Prognostic mortality factors such as MR and severity of LVEF decline were found to be relevant among patients presented with cardiogenic shock [13]. These previous findings do not correlate with our findings as described above, as none of our examined echo parameters were statistically significant to match the study outcome, although improved LVEF was connected to female sex and thrombolytic therapy, as opposed to renal failure and background hypertension. It might be related to a relatively small sample size, as unfortunately we found that a portion of low-LVEF post-STEMI patients were lost to follow-up, either in community clinics or even at our institutions’ out-patient clinic, although they were instructed to continue their management with follow-up echocardiography according to the guidelines. There was also a significantly lower portion of “LVEF-recoverees” compared to previous studies, as there might be other influencing co-factors such as significant difference in echo study quality between in-house index hospitalization study and the follow-up echo being performed in numerous echo labs in the south of Israel.

As for other echocardiographic features rather than LVEF delta, Moslah et al. found that end-diastolic wall stress, featured as a tool to assess myocardial remodeling after STEMI, was a predictor of MACE and longer hospitalizations, independent of LVEF [14], while other studies showed that global longitudinal strain (GLS) imaging predicted arrhythmic events post-STEMI independent of LVEF [9,15]. Our study did not find a predictable correlation between the echo parameters and LVEF recovery.

As for our second finding, it was demonstrated by Baron et al. that one year after MI, GLS significantly improved in patients with initially both normal and impaired EF, meaning that the depth of LVEF acute decline post-STEMI did not correlate with rates of recovery. However, initial impairment of LV function (by EF, WMSI or GLS), male gender, non-smoking and treatment with beta-blockers were independent predictors of GLS improvement in that study [16]. This partially correlates with our results, and it will be valuable to seek further information in future studies on the parameters that influenced the patients that did recover in our population and if good clinical and imaging follow-up can contribute to these results rather than the factors mentioned above.

While our research is primarily focused on the prognostic value of echo parameters, it is worth mentioning that, recently, platelet count and mean platelet volume (MPV) were shown to correlate to “all cause death” and MACE following ACS [17,18]. Exploring combined echo and hematological features in future studies may yield even more significant results.

SGLT2i therapy was initiated among 16 (15%) patients and across groups. We believe that this low percentage derives from the cohort years (2016–2021), when at the beginning of this period SGLT2i treatment was not widespread nor used as a first-line therapy for heart failure; hence, a similar prospective study would probably initiate different percentages.

As described briefly earlier, we also saw in our inclusion criteria that there is a significant portion of post-STEMI patients that were unfortunately lost to follow-up in Israels’ community services, despite specific indications and discharge letter instruction to perform a follow-up echocardiography study. Chew et al. have addressed this issue, stating that targeting processes affecting low rates of LVEF reassessment may reduce missed care opportunities and ensure that patients consistently receive appropriate evidence-based and guideline-recommended care [4].

Our study has a few limitations: First, although a tertiary, large and diversely populated referral medical center, this is still a single-center study. Second, the data were extracted retrospectively. Third, there were a number of subjects that we could not extract previous data about regarding their baseline ejection fraction and therefore might not show improvement as other previously pre-STEMI “normal” ejection fractions. Lastly, in comparison to previous data, it seems that our cohort size was not large enough and therefore not powerful enough to show a statistical trend towards some prognostic factors as described in previous papers.

## 6. Conclusions

In the population of STEMI patients with residual LVEF < 35%, approximately only a quarter will improve at least 10% in their follow-up LVEF, and there were no clear echocardiographic predictors for this improvement. In addition, the severity of LVEF decrease following the STEMI event was not associated directly with future improvement in follow-up LVEF. Furthermore, this study emphasizes the need for better follow-ups by community and out-patient cardiac clinics, in order to be stricter about both imaging and clinical treatment in this high-risk population of post-STEMI patients.

## Figures and Tables

**Table 1:** Change in LVEF across clinical parameters.

	<10% Improvement (*n* = 74)	>10% Improvement (*n* = 27)	Total (*n* = 101)	*p* Value
LVEF change (%)	1.25 (−5, 5)	20 (15, 25)	5 (−2.5, 12.5)	
Age	65 (53, 72)	61 (53, 71)	63 (53, 72)	0.61
Female sex	7 (9.5%)	8 (29.6%)	15 (14.9%)	0.01
Hospitalization length (days)	5 (3, 6)	4 (3, 6)	4 (3, 6)	0.88
Thrombolysis	3 (4.1%)	3 (14.8%)	7 (6.9%)	0.06
BMI	26.3 (23, 29.3)	25.7 (22.5, 30)	25.8 (23, 29.3)	0.95
Diabetes Mellitus	33 (44.6%)	10(37.0%)	43 (42.6%)	0.5
CHF	7 (9.50%)	1 (3.7%)	8 (7.90%)	0.34
MI	20 (27.0%)	12 (44.4%)	32 (31.7%)	0.1
PCI	19 (25.7%)	11 (40.7%)	30 (29.7%)	0.14
CABG	4 (5.40%)	0 (0.0%)	4 (4.00%)	0.11
CVA	2 (2.70%)	0 (0.0%)	2 (2.00%)	0.4
AF	3 (4.10%)	1 (3.7%)	4 (4.00%)	1
Smoker	37 (50.0%)	15 (55.6%)	52 (51.5%)	0.62
CPR	1 (1.40%)	1 (3.7%)	2 (2.00%)	0.46
IHD	18 (24.3%)	11 (40.7%)	29 (28.7%)	0.11
HTN	42 (56.8%)	9 (33.3%)	51 (50.5%)	0.9
OBESITY	8 (10.8%)	6 (22.2%)	14 (13.9%)	0.14
DYSLIPIDEMIA	46 (62.2%)	15 (55.6%)	61 (60.4%)	0.55
COPD	4 (5.40%)	2 (7.4%)	6 (5.90%)	0.7
PE	2 (2.70%)	0 (0.0%)	2 (2.00%)	1
ARF	0 (0.00%)	1 (3.7%)	1 (1.00%)	0.27
CRF	12 (16.2%)	0 (0.0%)	12 (11.9%)	0.026

In columns: percentage or 25–75 percentile. LVEF = left ventricle ejection fraction; BMI = body mass index; CHF = congestive heart failure; MI = myocardial infarction; PCI = percutaneous coronary intervention; CABG = coronary artery bypass graft; CVA = cerebral vascular accident; AF = atrial fibrillation; CPR = cardiopulmonary resuscitation; IHD = ischemic heart disease; HTN = hypertension; COPD = chronic obstructive pulmonary disease; PE = pulmonary embolism; ARF = acute renal failure; CRF = chronic renal failure.

**Table 2:** Changes in left ventricle ejection fraction (LVEF) across echocardiographic parameters.

	<10% Improvement (*n* = 74)	>10% Improvement (*n* = 27)	Total (*n* = 101)
LVEDD (cm)	4.9 (3.5, 5.2)	4.7 (4.4, 5)	4.8 (4.5, 5.2)
LVESD (cm)	3.6 (3.1, 4.2)	3.3 (3.0, 3.9)	3.5 (3.1, 4.1)
LAV (mL)	50 (38, 64)	45 (38, 59)	50 (38, 61)
LVEF	30 (25, 35)	25 (20, 35)	30 (22.5, 35)
2D_GLS	−10 (−11, −9)	−11 (−13, −10)	−10 (−11, −9)
E wave velocity	0.6 (0.51, 0.77)	0.56 (0.49, 0.63)	0.59 (0.51, 0.7)
A wave velocity	0.62 (0.49, 0.78)	0.68 (0.47, 0.86)	0.64 (0.49, 0.8)
E/e’ ratio	9.5 (8, 12.7)	9.3 (6.9, 10.8)	9.3 (8, 12.3)
MR (≥moderate)	2 (2.70%)	0 (3.7%)	1 (1.00%)
TR (≥moderate)	1 (1.40%)	10 (37.0%)	31 (30.7%)
AR (≥moderate)	0 (0%)	0 (0%)	0 (0%)
AS (≥moderate)	1 (1.40%)	0 (0.0%)	1 (1.00%)
sPAP	35 (29, 44)	31 (28, 36)	34 (28, 40)
RV dysfunction	15 (20.5%)	5 (18.5%)	20 (20.0%)
Diastolic function-normal	9 (12.2%)	0 (0.0%)	9 (8.90%)
Diastolic dysfunction-Grade I	18 (24.3%)	7 (25.9%)	25 (24.8%)
Diastolic dysfunction-grade II	16 (21.6%)	6 (22.2%)	22 (21.8%)
Diastolic dysfunction-Grade III	6 (8.10%)	0 (0.0%)	6 (5.90%)
Diastolic function-Undermined	1 (1.40%)	2 (7.4%)	3 (3.00%)
pericardial effusion	17 (23.0%)	3 (11.1%)	20 (19.8%)
LV thrombus	2 (2.70%)	0 (0.0%)	2 (2.00%)

In columns: percentage or 25–75 percentile. LVEDD = left ventricle end diastolic diameter; LVESD = left ventricle end systolic diameter; LAV = left atrium volume; LVEF = left ventricle ejection fraction; GLS = global longitudinal strain; MR = mitral regurgitation; TR = tricuspid regurgitation; AS = aortic stenosis; AR = aortic regurgitation; sPAP = systolic pulmonary artery pressure; RV = right ventricle.

**Table 3:** Laboratory data from index hospitalization and main medical treatment.

	<10% Improvement (*n* = 74)	>10% Improvement (*n* = 27)	Total (*n* = 101)	Significance
Alkaline Phosphatase	79 (67, 97.5)	83 (68, 113)	80.5 (68, 98)	0.5
Alanine Transaminase (ALT)	37 (23, 69)	42 (25, 90)	39 (23, 77)	0.37
Aspartate Aminotransferase(AST)	145 (35, 340)	118 (50, 182)	135 (38, 274)	0.28
Total Bilirubin	(0.59, 1.19)	0.535 (0.44, 0.7)	0.68 (0.51, 1)	0
Urea	0.79 (32.6, 50.7)	34.8 (30.6, 40.4)	37.8 (31.4, 47.2)	0.034
Highly sensitive C-Reactive Protein	40.1 (0.475, 8.745)	1.36 (0.62, 11.24)	1.53 (0.58, 8.97)	0.55
GammaGlutamyl Transferase	1.765 (18.5, 45.5)	37 (22, 57)	30 (19, 48)	0.09
Glucose	26.5 (123, 200)	154 (122, 184)	154 (122.5, 194.5)	0.78
Hemoglobin	154 (12.8, 15.1)	13.55 (12.5, 14.3)	14 (12.8, 14.9)	0.24
Hemoglobin A1C	14.1 (5.5, 7.5)	5.95 (5.5, 7)	6 (5.5, 7.5)	0.77
Lactate Dehydrogenase	6 (602, 1912)	953.5 (657, 1230)	1081.5 (633, 1801)	0.29
Partial Thromboplastin Time	1176.5 (30.3, 91.6)	33.6 (29.7, 43)	38.6 (29.95, 81.4)	0.18
Platelets	47.9 (186, 272)	240 (180, 310)	228.5 (185.5, 276)	0.2
Potassium	226 (3.9, 4.4)	4.1 (3.7, 4.3)	4.1 (3.85, 4.4)	0.62
Albumin	4 (3.3, 3.8)	3.55 (3.3, 3.9)	3.6 (3.3, 3.8)	0.6
Creatinine	3.6 (0.81, 1.19)	0.77 (0.57, 0.92)	0.9 (0.75, 1.15)	<0.001
Sodium	0.94 (137, 140)	139 (137, 140)	139 (137, 140)	0.88
White BloodCells	774.3 (8.93, 14.59)	11.61 (8.95, 14.17)	11.58 (8.95, 14.31)	**0.85**
Troponin T (max)	11.475 (2558.5, 6397)	1774 (746.2, 4334)	3843 (1530, 6228)	**0.013**
Norepinephrine	3 (4)	4 (15.3)	7 (7)	**0.05**
Beta blockers	68 (91.8)	24 (92.3)	92 (92)	**0.95**
Calcium Channel Blockers	4 (5.4)	0 (0)	4 (4)	**0.57**
Renin–Angiotensin–Aldosterone System inhibitor	65 (87.8)	23 (88.4)	88 (88)	**0.93**
Statins	71 (95.9)	25 (96.1)	96 (96)	**0.96**
Sodium Glucose co Transporter 2 inhibitor	12 (16.2)	4 (15.3)	16 (16)	**0.92**
Spironolactone	20 (27)	19 (70)	39 (37%)	**0.05**

**Table 4:** Propensity score adjustment.

Logistic Regression				Propensity Adjusted Ors *		
Variable	Univariate					
	OR	95% CI	*p* Value	OR	95% CI	*p* Value
Age	0.99	0.96–10.3	0.79			
Sex	4.03	1.3–12.5	0.016			
Ethnicity (Arab)	0.71	0.23–2.14	0.54			
Thrombolysis	4.1	0.86–19.77	0.08	1.98	0.34–11.5	0.45
Diabetes Mellitus	0.73	0.29–1.81	0.5			
CHF	0.37	0.43–3.14	0.36			
MI	2.16	0.86–5.4	0.1			
HTN	0.38	0.15–0.96	0.04			
Obesity	2.46	0.73–7.57	0.15			
IHD	2.14	0.84–5.44	0.11			
LVEF	0.96	0.91–1.02	0.15	0.93	0.87–0.99	0.035
LVEDD	0.5	0.21–1.16	0.11	0.43	0.15–1.17	0.099
LVESD	0.69	0.36–1.32	0.26	0.71	0.33–1.51	0.37
E velocity	0.07	0.002–2.84	0.16	0.03	0.001–2.13	0.11
A velocity	3.7	0.27–50.99	0.33	2.14	0.12–37.0	0.6
E/e’	0.94	0.83–1.07	0.35	0.95	0.82–1.11	0.51
RV dysfunction	0.99	0.41–2.4	0.99	1.43	0.52–3.98	0.49
Total bilirubin	0.092	0.016–0.512	0.006	0.125	0.02–0.73	0.021
WBC	0.97	0.90–1.05	0.51	0.99	0.91–1.08	0.86
Hemoglobin	0.96	0.77–1.21	0.75	0.97	0.75–1.24	0.79
Creatinine	0.085	0.012–0.62	0.015	0.27	0.04–2.11	0.21
Urea	0.96	0.924–0.997	0.032	0.96	0.92–1.005	0.085
C-Reactive Protein	1.012	0.987–1.037	0.35	1.02	0.99–1.05	0.26
Beta Blockers	1.06	0.2–5.6	0.95	1.07	0.16–7.20	0.94
Statins	1.06	0.11–10.63	0.96	3.27	0.21–50.03	0.39
Renin–Angiotensin–Aldosterone System inhibitor	1.06	0.26–4.3	0.93	0.55	0.12–2.5	0.44
Sodium Glucose co Transporter 2 inhibitor	0.94	0.27–3.22	0.92	1.09	0.27–4.41	0.9
PCI	1.99	0.79–5.03	0.15	0.81	0.25–2.6	0.73

CHF = congestive heart failure; MI = myocardial infarction; HTN = hypertension; IHD = ischemic heart disease; LVEF = left ventricle ejection fraction; LVEDD = left ventricle end diastolic diameter; LVESD = left ventricle end systolic diameter; PCI = percutaneous coronary intervention. * Propensity score was based on the following variables: age, sex, ethnicity, diabetes mellitus, hypertension, congestive heart failure, prior myocardial infarction, smoking status, obesity and days of follow-up.

**Table 5:** Primary catheterization data according to change in LVEF (%).

		LVEF Change Less than 10%					LVEF Improved by More than 10%					Total					*p* Value
		Median	Percentile 25	Percentile 75	Count	Column N %	Median	Percentile 25	Percentile 75	Count	Column N %	Median	Percentile 25	Percentile 75	Count	Column N %	
Time to Balloon_minutes		63	45	85			75	56	82			66	47	84			0.13
Culprit Coronary Artery of index infarction	LAD				48	64.9%				19	70.4%				67	66.3%	0.6
	LCX				10	13.5%				4	14.8%				14	13.9%	
	RCA				15	20.3%				3	11.1%				18	17.8%	
	LM				1	1.4%				1	3.7%				2	2.0%	
Coronary Arteries with significant disease > 50% stenosis or positive FFR	1				19	25.7%				8	29.6%				27	26.7%	0.1
	2				22	29.7%				13	48.1%				35	34.7%	
	3				33	44.6%				6	22.2%				39	38.6%	
Coronary Arteries revascularize during index procedure	0				11	14.9%				2	7.4%				13	12.9%	0.29
	1				59	79.7%				23	85.2%				82	81.2%	
	2				3	4.1%				1	3.7%				4	4.0%	
	3				1	1.4%				1	3.7%				2	2.0%	
Staged PCI	No				28	39.4%				14	56.0%				42	43.8%	0.15
	Yes				43	60.6%				11	44.0%				54	56.3%	

LVEF = left ventricle ejection fraction; LAD = left anterior descending; LCX = left circumflex artery; RCA = right coronary artery; LM = left main coronary artery; FFR = fractional flow reserve; PCI = percutaneous coronary intervention.

## Data Availability

The data that support the findings of this study are available from the corresponding author upon reasonable request.

## Abbreviation

ST	Elevation myocardial infarction (STEMI)
LV	Left ventricle
GLS	Global longitudinal strain
PCI	Percutaneous coronary intervention
CHF	Congestive heart failure
CICU	Cardiac Intensive Care Unit
IVS	Interventricular septum
LVPW	Left ventricle posterior wall
LA	Left atrium
LVEF	Left ventricle ejection fraction
MR	Mitral regurgitation
TR	Tricuspid regurgitation
AR	Aortic regurgitation
RA	Right atrium

## References

[1] Kim D.H., Park C.B., Jin E.S., Hwang H.J., Sohn I.S., Cho J.M., Kim C. (2018). Predictors of decreased left ventricular function subsequent to follow-up echocardiography after percutaneous coronary intervention following acute ST-elevation myocardial infarction. Exp. Ther. Med..

[2] Choudhury T., West N.E.J., El-Omar M. (2016). ST elevation myocardial infarction. Clin. Med. J. R Coll. Physicians Lond..

[3] Frampton J., Devries J.T., Welch T.D., Gersh B.J. (2020). Modern Management of ST-Segment Elevation Myocardial Infarction. Curr. Probl. Cardiol..

[4] Chew D.S., Wilton S.B., Kavanagh K., Southern D.A., Tan-Mesiatowsky L.E., Exner D.V. (2018). Left ventricular ejection fraction reassessment post–myocardial infarction: Current clinical practice and determinants of adverse remodeling. Am. Heart J..

[5] Brooks G.C., Lee B.K., Rao R., Lin F., Morin D.P., Zweibel S.L., Buxton A.E., Pletcher M.J., Vittinghoff E., Olgin J.E. (2016). Predicting Persistent Left Ventricular Dysfunction Following Myocardial Infarction the PREDICTS Study. J. Am. Coll. Cardiol..

[6] Garber L., McAndrew T.C., Chung E.S., Stancak B., Svendsen J.H., Monteiro J., Fischer T.M., Kueffer F., Ryan T., Bax J. (2018). Predictors of Left Ventricular Remodeling after Myocardial Infarction in Patients with a Patent Infarct Related Coronary Artery after Percutaneous Coronary Intervention (from the Post-Myocardial Infarction Remodeling Prevention Therapy [PRomPT] Trial). Am. J. Cardiol..

[7] Mollema S.A., Nucifora G., Bax J.J. (2009). Prognostic value of echocardiography after acute myocardial infarction. Heart.

[8] Abou R., Van Der Bijl P., Bax J.J., Delgado V. (2020). Global longitudinal strain: Clinical use and prognostic implications in contemporary practice. Heart.

[9] Haugaa K.H., Smedsrud M.K., Steen T., Kongsgaard E., Loennechen J.P., Skjaerpe T., Voigt J.-U., Willems R., Smith G., Smiseth O.A. (2010). Mechanical Dispersion Assessed by Myocardial Strain in Patients After Myocardial Infarction for Risk Prediction of Ventricular Arrhythmia. JACC Cardiovasc. Imaging.

[10] Biering-Sørensen T., Biering-Sørensen S.R., Olsen F.J., Sengeløv M., Jørgensen P.G., Mogelvang R., Shah A.M., Jensen J.S. (2019). Global Longitudinal Strain by Echocardiography Predicts Long- Term Risk of Cardiovascular Morbidity and Mortality in a Low Risk General Population: The Copenhagen City Heart Study. Physiol. Behav..

[11] Pokorney S.D., Miller A.L., Chen A.Y., Thomas L., Fonarow G.C., de Lemos J.A., Al-Khatib S.M., Peterson E.D., Wang T.Y. (2015). Implantable cardioverter-defibrillator use among medicare patients with low ejection fraction after acute myocardial infarction. JAMA—J. Am. Med. Assoc..

[12] Oh P.C., Choi I.S., Ahn T., Moon J., Park Y., Seo J.G., Suh S.Y., Ahn Y., Jeong M.H. (2013). Predictors of recovery of left ventricular systolic dysfunction after acute myocardial infarction: From the Korean acute myocardial infarction registry and Korean myocardial infarction registry. Korean Circ. J..

[13] Picard M.H., Davidoff R., Sleeper L.A., Mendes L.A., Thompson C.R., Dzavik V., Steingart R., Gin K., White H.D., Hochman J.S. (2003). Echocardiographic predictors of survival and response to early revascularization in cardiogenic shock. Circulation.

[14] Mosleh W., Elango K., Shah T., Chaudhari M., Gandhi S., Kattel S., Karki R., Khalil C., Frodey K., Dahal S. (2018). Elevated end-diastolic wall stress after acute myocardial infarction predicts adverse cardiovascular outcomes and longer hospital length of stay. Echocardiography.

[15] Haugaa K.H., Grenne B.L., Eek C.H., Ersbøll M., Valeur N., Svendsen J.H., Florian A., Sjøli B., Brunvand H., Køber L. (2013). Strain echocardiography improves risk prediction of ventricular arrhythmias after myocardial infarction. JACC Cardiovasc. Imaging.

[16] Baron T., Christersson C., Hjorthén G., Hedin E.M., Flachskampf F.A. (2018). Changes in global longitudinal strain and left ventricular ejection fraction during the first year after myocardial infarction: Results from a large consecutive cohort. Eur. Heart J. Cardiovasc. Imaging.

[17] Galimzhanov A., Sabitov Y., Tenekecioglu E., Tun H.N., Alasnag M., Mamas M.A. (2022). Baseline Platelet Count in Percutaneous Coronary Intervention: A Dose–Response Meta-Analysis. Heart.

[18] Galimzhanov A., Tenekecioglu E., Rustamova F., Tun H.N., Mamas M.A. (2022). The Prognostic Utility of Mean Platelet Volume in Patients with Acute Coronary Syndrome: A Systematic Review with Meta-Analyses. Angiology.

